# Modulating Thyroid Hormone Levels in Adult Mice: Impact on Behavior and Compensatory Brain Changes

**DOI:** 10.1155/2021/9960188

**Published:** 2021-06-24

**Authors:** Dana M. Niedowicz, Wang-Xia Wang, Doug A. Price, Peter T. Nelson

**Affiliations:** ^1^Sanders Brown Center on Aging, University of Kentucky, Lexington, KY 40536, USA; ^2^Department of Pathology, Division of Neuropathology, University of Kentucky, Lexington, KY 40536, USA

## Abstract

Thyroid hormone (TH) perturbation is a common medical problem. Because of substantial public health impact, prior researchers have studied hyper- and hypothyroidism in animal models. Although most prior research focused on *in utero* and/or developmental effects, changes in circulating TH levels are commonly seen in elderly individuals: approximately 20% of persons older than 80 years have clinically impactful hypothyroidism and up to 5% have clinical hyperthyroidism, with women being more often affected than men. TH disease model methodology in mice have varied but usually focus on a single sex, and the impact(s) of TH perturbation on the adult brain are not well understood. We administered thyroxine to middle-aged (13 to 14 months) male and female mice to model hyperthyroidism and TH-lowering drugs propylthiouracil (PTU) and methimazole, to induce hypothyroidism. These pharmacological agents are used commonly in adult humans. Circulating TH-level changes were observed when thyroxine was dosed at 20 *µ*g/mL in drinking water for two weeks. By contrast, PTU and methimazole did not elicit a consistent reproducible effect until two months of treatment. No substantial changes in TH levels were detected in brain tissues of treated animals; however, pronounced changes in gene expression, specifically for TH-processing transcripts, were observed following the treatment with thyroxine. Our study indicated a robust compensatory mechanism by which the brain tissue/cells minimize the TH fluctuation in CNS by altering gene expression. Neurobehavioral changes were related to the TH perturbation and suggested potential associations between cognitive status and hyper- and hypothyroidism.

## 1. Introduction

Thyroid hormone (TH) is a potent regulator of development and metabolism throughout the animal kingdom[1]. Thyroxine (tetraiodothyronine, or T4) is the iodine-containing prohormone released into the blood from the thyroid gland. The more bioactive form of TH is triiodothyronine (T3), formed from the enzymatic removal of an iodine, which subsequently complexes with intracellular nuclear receptors to affect gene transcription. T4 and T3 also exert effects via intracellular signalling [2].

Circulating and tissue levels of TH are maintained in a relatively narrow concentration range [3–6]. Alterations in blood TH levels can have detrimental effects in a variety of tissues, including heart, kidney, liver, and brain. Hyperthyroidism is commonly associated with weight loss, increased heart rate, thinning hair, insomnia, and other clinical manifestations. On the other hand, hypothyroidism in humans is associated with weight gain, fatigue, muscle weakness, and impaired memory. Although both conditions have proven treatments, the symptom can be subtle, and diagnosis may occur years after disease onset [7–9]. Although normalization of TH levels via pharmaceutical intervention leads to attenuation of many symptoms, some perturbations may be irreversible. Current guidelines focus on children and young adults primarily, taking into account the underlying etiology and treating accordingly [10, 11]. Specific guidance for clinical TH management in the elderly has been provided but is less clear and may be complicated by comorbidities and possible age-related changes in TH processing.

One of the tissues that exhibits the most profound changes in response to circulating TH perturbations is the brain. TH dysregulation has well-described effects on brain development in utero, with maternal deficiencies linked to developmental and cognitive delays [12, 13]. In addition, changes in circulating TH levels have pathological effects in adulthood [2, 6, 13–15]. Hypothyroidism in humans has been linked to both cognitive and motor defects [2]. Animal models have correlated some cognitive impairment to hippocampal alterations [13]. Furthermore, CSF and brain TH concentrations have been correlated with age-related neurodegenerative diseases, such as LATE (Limbic-predominant age-related TDP-43 encephalopathy) and cerebrovascular disease [16–19], suggesting long-term consequences to perturbations in these pathways.

Rodents have been used as experimental model systems to study the effects of TH dysfunction due to perturbations in utero, later development, and during adulthood. Yet published studies vary in the reported treatment conditions, including dose, treatment length, method of delivery, as well as strain and sex of experimental animals [15, 20–23]. Most prior studies of TH dysregulation in animal models only evaluated a single sex. Sex is an important parameter because TH disease in human aging is sexually dimorphic [16].

We performed a series of studies testing dose ranges and treatment length in adult mice to evaluate changes in circulating TH levels, as well as downstream effects on physiological and behavioral parameters, and also gene expression. We focused on global changes in the brain, using both male and female mice. This report details hyper- and hypothyroidism mouse modeling in aged animals and provides new evidence of the physiological and behavior effects of hyper- and hypothyroidism.

## 2. Materials and Methods

### 2.1. Animals

All animal work was conducted with the approval of the University of Kentucky IACUC and was performed in accordance with PHS guidelines. Aged (13-14 months) C57Bl/6J (Jax #000664) mice were obtained from the National Institute on Aging (Bethesda, MD), while young (2 months) mice were obtained from the Jackson Laboratory (Bar Harbor, ME). Mice were group housed, fed and watered *ad libitum*, and maintained on a constant, 14/10-hour light/dark cycle. Mice were euthanized by barbiturate overdose (SomnaSol™, Med-Pharmex Inc., Pomona, CA), followed by cardiac perfusion with PBS using a peristaltic pump (Fisher Scientific, Hampton, NH). The hearts were weighed immediately after perfusion without freezing. The brains were divided using a scalpel in the sagittal plane; half was fixed in 10% phosphate-buffered formalin, whereas the other was immediately frozen at −80°C after the removal of the cerebellum.

### 2.2. Treatments

In order to model hyperthyroidism, levothyroxine (Spectrum Chemical MFG Corp, New Brunswick, NJ) was administered in drinking water (2–20 *µ*g/mL). A 50X stock solution containing BSA (0.1%) and NaOH (4 mM) was refrigerated in the dark and diluted weekly for use. The hypothyroid model used both methimazole (Spectrum Chemical MFG Corp) in drinking water (0.02% w/v) and a commercially available, low-iodine chow containing propylthiouracil (PTU; 0.15% w/w; Teklad #TD.95125). Control mice were fed a control diet (Teklad #TD.97350) with the same nutrient content and provided water with no additives. All groups were split approximately equally between males and female mice. Mice were weighed daily for the first week of treatment, then weekly thereafter. After three months of treatment, food and water consumption was measured daily for two weeks. The amounts ingested were measured for each cage, then divided by the number of mice in the cage to obtain the average amount consumed per mouse. For a summary of animal numbers, ages, and assays/tests, see Supplementary Table 1.

### 2.3. Glucose Measurement

Blood glucose was analyzed at three months of treatment, measuring either near the start of the light cycle (nonfasting) or after a six-hour fast during the light cycle (fasting). A drop of blood was obtained by tail nick of live mice, and blood glucose measured by glucometer (Bayer Breeze 2: Bayer, Tarrytown, NJ) using compatible test strips.

### 2.4. Brain Extractions

Frozen hemibrains (no cerebellum) were homogenized in RIPA buffer containing protease inhibitor cocktail (Thermo Fisher #1860932: Waltham, MA) using a polytron homogenizer (PR0200, ProScientific, Oxford, CT). A small aliquot of homogenate was immediately extracted with TRIzol™ (Invitrogen, Waltham, MA) in order to obtain RNA for gene expression analyses (see below) and frozen at −80°C until use. For biochemical analyses, the RIPA homogenate was centrifuged to remove insoluble material (12,000x g, 15 min., 4°C), and the resulting extract was transferred to a fresh tube and frozen at −80°C until use.

### 2.5. TH Assays

Commercially available total T3 and T4 enzyme-linked immunosorbent assay kits (ELISA: Aviva Systems Biology, San Diego, CA) were used to assess TH levels in both serum and brain extracts. Blood was collected by saphenous bleed at baseline and at two weeks (for the dosage experiments), two months, and four months of treatment. In each case, serum was collected in the morning. The blood was allowed to coagulate for 20–30 minutes, then centrifuged at a low speed (1,500x g, 15 minutes at room temperature). Serum was transferred to a fresh tube and stored at −80°C. In order to maintain consistency with assay standards, which contain serum matrix proteins, the samples were diluted five-fold with the kit-supplied zero standard. All serum samples were diluted identically in order to reduce intersample dilution effects. Serum from all time points for the four-month treatment was analyzed on a single microplate and compared against the same standard curve. Serum from the dosage experiments (Supplementary Figure S1) was analyzed independently at the time of the blood draw, comparing against control animals each time, using its own standard curve. Brain RIPA extracts were analyzed separately from serum and were loaded neat. ELISAs were performed according to the manufacturer's instructions. Absorbance was measured at 450 nm using the SpectraMax M3 (Molecular Devices Inc., Sunnyvale, CA, USA) plate reader. Standard curves were generated using SigmaPlot software (Systat Software, San Jose, CA, USA), using a 4-parameter logistic fit, which was used to calculate T3 and T4 sample concentrations.

### 2.6. Behavioral Tests

All behavioral tests were performed in the University of Kentucky Rodent Behavioral Core facility, a space dedicated to this testing exclusively, in order to minimize outside noise and disruption. All tests were performed in accordance with NIH animal welfare policies and with prior IACUC approval. Open field testing was performed at two and four months of treatment using a multichamber open field arena (SD Instruments, San Diego, CA). Briefly, mice were placed in an empty arena, and their movement was recorded for 15 minutes. In order to reduce habituation effects, the arena in which the mice were placed was rotated for all mice at four months. Elevated plus maze was used only at four months to augment the open field data without the confounding effect of habituation. Briefly, mice were placed in the center of the elevated plus maze apparatus (Noldus, Leesburg, VA), and their movement was tracked for five minutes. Data for both tests were captured and analyzed using Ethovision XT 8.0 video-tracking software (Noldus).

### 2.7. Gene Expression

Brain TRIzol™ extracts were subjected to a phenol-chloroform purification. The resulting RNA was analyzed for quantity and purity by Nanostring Spectrophotometer (ND-1000, NanoDrop Technologies, Wilmington, DE), then DNase treated, and reverse transcribed to obtain cDNA (Superscript, Applied Biosystems). Expression of a select set of genes was analyzed with custom-designed gene array card (Applied Biosystems #4342253) using TaqMan™ Universal PCR Master Mix (Applied Biosystems). Assay IDs for each gene are listed in Supplementary Table S2. Changes in gene expression were calculated using the comparative *C*_*T*_method, standardizing the threshold cycle to the geometric mean of two housekeeping genes (*Sdha* and *Pgk*1), then comparing each treatment group to control-treated mice [24–26].

### 2.8. Data Analyses and Statistical Tests

Each mouse's data were assembled individually, then used to calculate group averages. Data were then reported as group mean ± SEM. The exception to this was the food and water consumption, which were obtained as cage averages, divided by the number of mice per cage. Data were analyzed for statistical significance with SPSS (Hewlett Packard; Palo Alto, CA) using the general linear model (GLM) module with ANOVA for the independent variables (as needed) of treatment or time point . For an explanation of this model, see https://www.ibm.com/support/knowledgecenter/SSLVMB_25.0.0/statistics_mainhelp_ddita/spss/advanced/idh_glm_multivariate.html.

## 3. Results

### 3.1. Treatment Parameters

A range of experimental conditions were evaluated and optimized, including changes of drug dosage and mode of delivery. We initially tested levothyroxine treatment using 2-month-old (young adult) mice (Supplementary Figure S1A). Both male and female mice were used, and compounds utilized in the study are commonly used to treat human disease. Low-thyroxine dosages (2–4 *µ*g/mL) did not consistently increase detectable circulating TH levels after a two-week treatment (there was a trend with *p* = 0.02 − 0.09; Supplementary Figure S1B). Increasing the dose to 20 *µ*g/mL in drinking water led to a robust increase in circulating T4 by two weeks of treatment (*p* < 0.00004). Because our focus was on aged mice, we reproduced these results in 13-month-old (analogous to human middle age) mice (*p* = 0.01; Supplementary Figure S1C). We initially administered PTU (0.15% w/v) in drinking water, with or without Kool-Aid flavoring to mask the taste of the drug. Because a large number of the mice declined to drink the PTU-containing water, we changed to a commercially available chow that contains PTU (0.15% w/w), and which has a reduced iodine content. We also augmented the effect of PTU by adding a small amount of methimazole (0.02% w/v) to drinking water. Despite more reliable consumption of the chow and treated water, there was still not substantial suppression of circulating T4 after two weeks of treatment (*p* = 0.27; Supplementary Figure S1C). Although the amount of PTU in the chow was not changed, we tried to increase the methimazole dose. The mice declined to drink the higher doses, however (*data not shown*). Therefore, we extended the treatment period to two and, ultimately, four months, maintaining the same treatment paradigm (see below).

**Figure 1:**
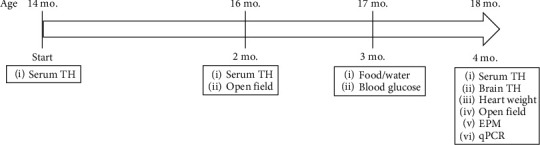
Timeline and schematic summary of the experiments. Middle-aged mice were treated with either levothyroxine (TH, 20 *µ*g/mL) or a combination of PTU (0.15%) and methimazole (0.02%) starting at 14 months and continuing for 4 months. Body weight was measured at least weekly throughout. Serum TH was measured at baseline, 2 months, and 4 months. General activity was tested at 2 and 4 months. Blood glucose and water/food consumption were determined at 3 months of treatment. At endpoint, tissue weights, brain TH, and gene expression were analyzed.

**Figure 2:**
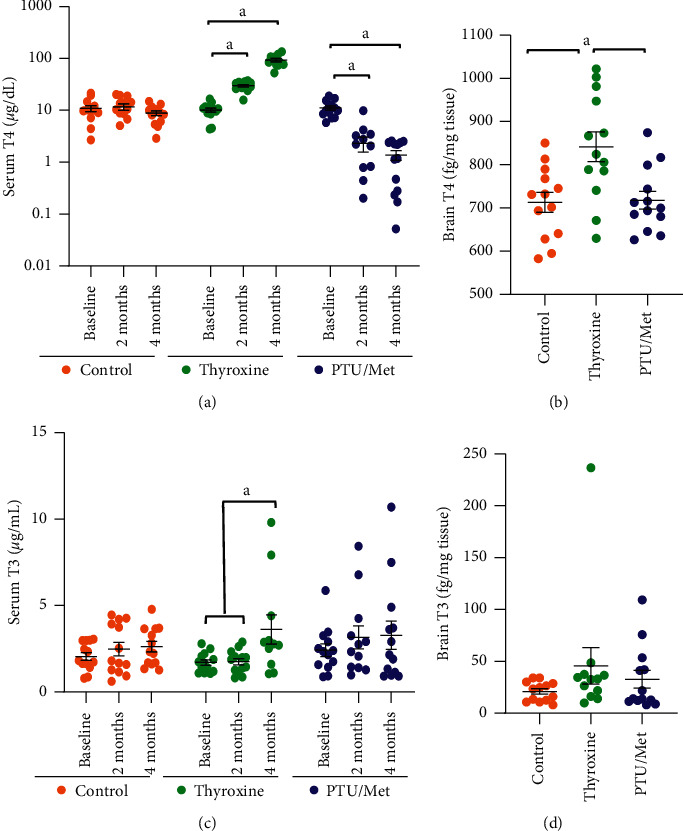
Serum and tissue levels of T4. Wild-type mice were treated with levothyroxine or a PTU/Met combination for four months, starting at 14 months old. (a) Thyroxine treatment led to a large increase in serum T4 by two months, then increased even further by four months. There was a significant decrease in circulating T4 in animals treated with PTU/Met by two months. This was maintained at four months, although there was no additional change. Note that this graph has a logarithmic *y*-axis. (b) There was a small increase in brain T4 in thyroxine-treated mice, but PTU/Met treatment did not have an effect. (c) Unlike T4, there was no significant change in serum T3 at two months, although there was a small increase at four months with thyroxine treatment. PTU treatment did not lead to a change at either time point. Brain levels of T3 (d) were unchanged in both treatment groups. Control = 7 M/6 F, thyroxine = 5 M/5 F (heart), and PTU/Met = 5 M/8 F. ^*a*^*p* ≤ 0.05.

Using the system of treatments described above, we carried out additional experiments in a larger set of middle-aged mice (*N* = 13 per group), testing the effects on serum and tissue TH, heart and body weight, blood glucose, general activity, and brain gene expression (Figure 1). As seen previously, there was a large increase in serum T4 by two months of thyroxine treatment (*p* = 0.001), which increased further by four months (*p* ≤ 0.0005; Figure 2(a)). PTU/methimazole (PTU/Met) treatment, on the other hand, significantly reduced circulating T4 by two months (*p* < 0.0005), and then the effect plateaued (*p* = 0.41 for the comparison between two and four months).

### 3.2. Tissue TH Levels

Given our interest in the effect of TH perturbations on the adult mammalian brain, we next wanted to know whether the observed changes in circulating T4 were associated with variation in TH levels in brain. Although thyroxine treatment led to a ten-fold increase in serum T4, there was a much smaller, but significant, increase in brain T4 (less than 1.2-fold; *p* = 0.01; Figure 2(b)). There was no change in brain T4 levels in PTU/Met-treated mice, despite a nearly seven-fold drop in circulating T4 levels (*p* = 0.99). These data underscore that the brain has robust compensatory mechanisms to maintain homeostatic levels of brain TH. Notably, although the scale of change in serum T4 was much greater than that in the brain tissue, there was a strong, positive correlation between the two measures. This was true at both two months (Supplementary Figure S2A; *p* = 0.001) and four months (Supplementary Figure S2B; *p* < 0.0001), indicating that the amount of TH that is present in the brain is impacted by circulating T4 levels. Furthermore, the ELISA showed consistent trends in the same animals, whether we were testing sera from live animals or frozen tissue samples extracted using RIPA buffer.

T3 is the more (relative to T4) bioactive form of TH, circulating at a much lower concentration, and it is formed predominantly within end-organ cells. Serum T3 levels were unchanged at two months of either TH or PTU/Met treatment (*p* ≥ 0.7; Figure 2(c)). Thyroxine treatment did lead to a small increase in serum T3 at four months (*p* = 0.02); however, PTU/Met had no detectable effect at the same time point (*p* = 0.61). Similarly, there was no significant change in brain T3 level, relative to controls, following either treatment (*p* ≥ 0.37; Figure 2(d)).

### 3.3. Other Outcomes

**Figure 3:**
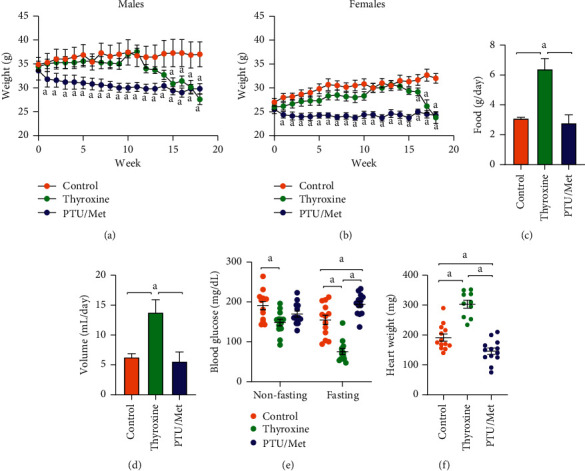
Effects of the treatments on food/water consumption, body weight, and blood glucose levels. Body weight was monitored in both males (a) and females (b) during the course of treatment. Thyroxine treatment did not affect weight until after 15 weeks of treatment, after which the mice quickly lost weight. PTU/Met-treated mice lost a bit of weight in the first week, then maintained their weight for the duration of the study. Food (c) and water (d) consumption were significantly increased in thyroxine-treated mice (at three months of treatment). *N* = 4 cages/treatment group. (e) Both fasting and nonfasting blood glucose levels were significantly lower in thyroxine-treated mice. Fasting blood glucose was increased in PTU/Met-treated mice, although nonfasting glucose was unaffected. (f) Heart weight was significantly increased in thyroxine-treated mice and decreased in PTU/Met-treated mice.  ^*∗*^*p* ≤ 0.05compared with control animals. Control = 7 M/6 F, thyroxine = 5 M/5 F (heart), and PTU/Met = 5 M/8 F. ^*a*^*p* ≤ 0.05.

Mice treated with PTU/Met weighed less than control mice at almost all time points after baseline weight measurement (Figures 3(a)and 3(b)). The weight loss in this group started within the first week of treatment, then plateaued for the remainder of the study, and it may be due to initial resistance to the flavor of the treatment chemicals in chow and water. Hyperthyroid mice did not lose significant weight until 15 (males; *p* ≤ 0.04) to 16 (females; *p* ≤ 0.08) weeks of treatment, then lost weight precipitously (Figures 3(a)and 3(b)). Despite eating and drinking about twice as much as control and PTU/Met-treated mice (*p* ≤ 0.008; Figures 3(c)and 3(d)), the weight loss in thyroxine-treated mice was pronounced by three months of treatment. There was no detected effect on food and water consumption in the PTU/Met-treated mice (*p* ≥ 0.73).

Because increased food and water consumption are known symptoms of metabolic syndrome and diabetes mellitus, which also have been linked to TH perturbations, we measured blood glucose in the mice. After three months, thyroxine treatment led to a decrease in both nonfasting and fasting blood glucose levels (*p* ≤ 0.04; Figure 3(e)); thus, the other changes did not correlate with hyperglycemia. PTU/Met treatment did not affect nonfasting blood glucose levels (*p* = 0.2) but led to an increase in fasting glucose levels (*p* = 0.002).

Cardiac hypertrophy is a well-established consequence of hyperthyroidism in humans [27]. We saw substantial heart weight increase in mice treated with thyroxine (*p* ≤ 0.0005; Figure 3(f)): their heart weighed almost twice as much as those from control-treated mice after four months of treatment. By contrast, PTU/Met-treatment led to a significant reduction in heart weight (*p* = 0.003).

### 3.4. Behavioral Tests

**Figure 4:**
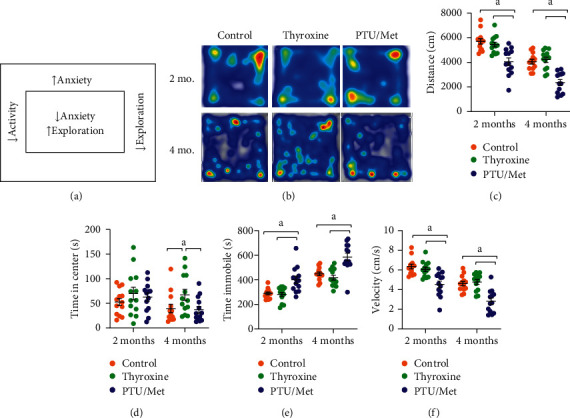
Open field tests of treated mice. Open field was performed at two and four months of treatment. (a) The open field arena had two main zones for analysis—the center and periphery. Animals with reduced anxiety and increased exploration spent more time in the center than those with more anxiety. (b) Representative raw data from the open field testing for each group. (c) All treatment groups displayed reduced movement at four months compared with two months. Thyroxine treatment did not have a significant effect on the distance moved at either time point. PTU/Met treatment led to a decrease in the distance moved at both two and four months. (d) There was no difference in the time spent in the center for any treatment group at two months of treatment. After four months, thyroxine-treated mice spent significantly more time in the center. (e) PTU/Met-treated mice spent more time immobile at both two and four months than either the control or thyroxine-treated mice. (f) PTU/Met-treated mice moved at a significantly lower velocity. ^*a*^*p* ≤ 0.05compared with control. Control = 7 M/6 F, thyroxine = 7 M/6 F, and PTU/Met = 5 M/8 F.

Another characteristic of TH dysfunction in humans is a change in activity level: hypothyroid individuals are generally more sedentary, whereas hyperthyroid humans display higher activity [6]. We used two different behavioral tests to assess general activity and as a neurobehavioral model of anxiety: open field and the elevated plus maze (EPM). In the open field test, animals with behavioral changes linked to anxiety and/or reduced exploration spend more time in the periphery of the empty arena, whereas those with lower anxiety spend more time in the middle (Figure 4(a)). Thyroxine treatment was not associated with change in the distance moved during open field testing compared with control-treated mice, at either two or four months of treatment (*p* ≥ 46; Figures 4(b)and 4(c)). PTU/Met-treated mice were significantly less active during the open field test than both control and thyroxine-treated mice after two months of treatment (*p* ≤ 0.0005). The same effect was observed at four months (*p* ≤ 0.0005). Additionally, PTU/Met-treated mice spent more time immobile (Figure 4(e)) and moved more slowly (Figure 4(f)) than the other two treatment groups. It should be noted that activity at four months was reduced from the two-month levels in all mice regardless of the treatments.

**Figure 5:**
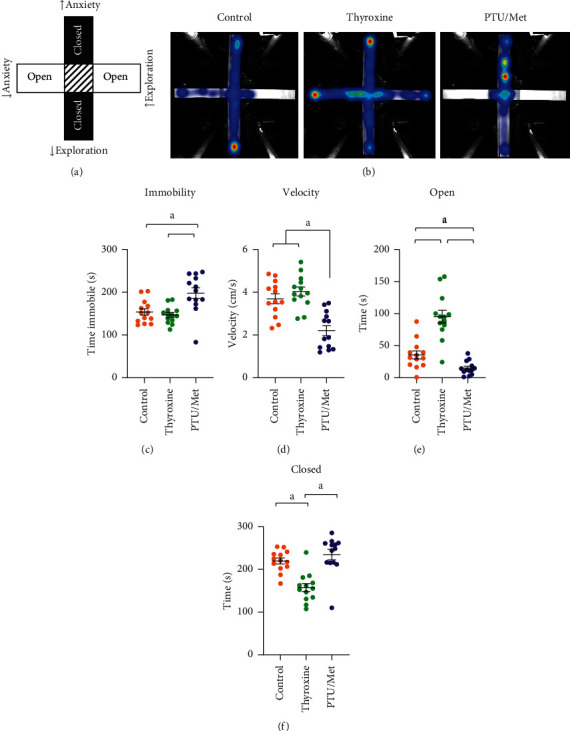
Elevated plus maze (EPM) test of treated mice. EPM was performed at four months of treatment. (a) Animals with apparent low anxiety and increased exploration will spend more time in the open arms while those with increased anxiety spend more time in the closed arms. (b) Representative raw data are shown for each treatment group. (c) Although thyroxine treatment had no effect on immobility during the elevated plus maze, PTU/Met-treated mice spent more time immobile. (d) PTU/Met-treated mice moved at a significantly lower velocity than either control or thyroxine-treated mice. (e) Thyroxine-treated mice spent significantly more time in the open arm, whereas PTU/Met-treated mice spent significantly less time than control mice. (f) Thyroxine-treated mice spent less time in the closed arms. There was no difference between PTU/Met and control-treated mice. ^*a*^*p* ≤ 0.05compared with control. Control = 7 M/6 F, thyroxine = 7 M/6 F, and PTU = 5 M/8 F.

Because it was unclear whether the reduction in activity between two and four months was due to test habituation or age, we performed EPM only at four months. This maze is used to test many of the same behaviors as the open field, with exploration of the open arms indicating reduced anxiety (Figure 5(a)). Additionally, we were able to track the amount of time spent moving and immobile as a measure of general activity. PTU/Met-treated mice spent significantly more time immobile during the elevated plus maze than control mice (*p* = 0.002; Figures 5(b)and 5(c)) and had a lower speed (Figure 5(d)) consistent with the lower activity that was observed during open field test.

These behavioral tests are used to model changes in anxiety and to measure presumed exploratory behavior. In the open field test, this is partly measured as the amount of time spent in the center part of the arena. There was no change in this parameter in either treatment group at two months of treatment, but thyroxine-treated mice spent significantly more time in the center at four months (*p* = 0.037; Figure 4(d)). Similarly, this treatment group spent significantly more time in the open arms (*p* ≤ 0.0005) and less in the closed arms (*p* ≤ 0.0005) during the elevated plus maze (Figures 5(e)and 5(f)). PTU/Met-treated mice spent significantly less time in the open arms than the controls during the elevated plus maze (*p* = 0.043).

### 3.5. Gene Expression

**Figure 6:**
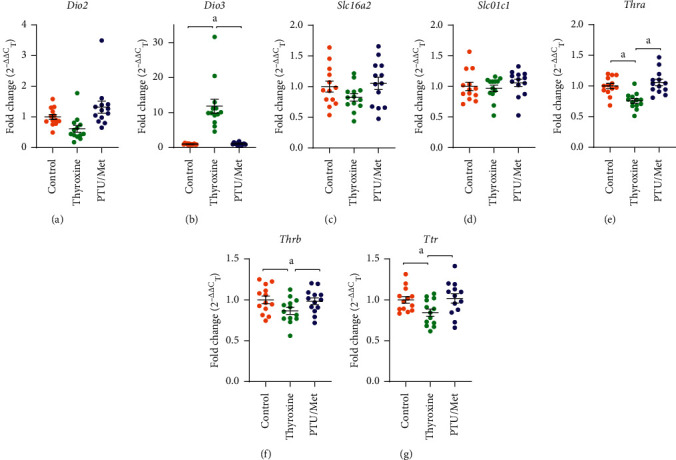
Gene expression following TH pertubation. RNA was extracted from brain and the effect of treatment on gene expression of a select set of genes was tested by qPCR. Several genes related to TH activation/inactivation, transport, and use were significantly affected by thyroxine treatment. (a) *Dio*2 trended toward a decrease with thyroxine treatment, whereas the inactivating *Dio*3 was robustly expressed (b). Expression of the two brain-specific TH transporters, *Slc*16*a*2 (c) and *Slc*01*c*1 (d) were not significantly changed by either treatment. Expression of both TH receptors (*Thra* (e) and *Thrb* (f)) was reduced compared with control-treated mice. (g) Transthyretin (*Ttr*) expression was also reduced in thyroxine-treated mice. Although there were not significant changes in expression of these genes with PTU/Met treatment, *Dio*2 expression tends to increase compared with control. ^*a*^*p* ≤ 0.05compared with control. The dashed line represents the control group, all standardized to 1. Control = 7 M/6 F, thyroxine = 7 M/6 F, and PTU/Met = 5 M/8 F.

Analyzing gene expression levels in mice brains, we compared specific transcripts in the brains of TH- and PTU/Met-treated mice with controls. Substantial differences were observed for genes known to process TH (Figure 6(a)–6(g)). These genes encode for proteins that are responsible for (1) transport of T3 and T4 into the brain, (2) activation and deactivation of both forms of the hormone, and (3) receptors that mediate downstream gene expression.

PTU/Met treatment did not generate much detectable effect, except the expression of *Dio*2 trended toward an increase in expression in these mouse brains (*p* = 0.11; Figure 6(a)). Thyroxine treatment elicited a stronger set of effects. The expression of *Dio*3, the thyroxine inactivating enzyme, robustly increased (*p* ≤ 0.0005; Figure 6(b)), whereas the expression of *Dio*2, the activating enzyme, tended to be decreased in these mice (*p* < 0.06). There was little to no change in the T3 and T4 transporters (*Slc*16*a*2 and *Slc*01*c*1; *p* = 0.151 and *p* = 0.711, respectively: Figures 6(c)and 6(d)). Expression of the TH receptors, *Thra* and *Thrb*, were decreased (*p* ≤ 0.0005and *p* = 0.032, respectively; Figures 6(e)and 6(f)), and transthyretin (*Ttr*) expression was also reduced (*p* = 0.027; Figure 6(g)).

## 4. Discussion

### 4.1. Mice vs. Humans

Our data emphasize the need to carefully consider the treatment parameters used when investigating the impact(s) of TH dysregulation in mice. We expected to easily reproduce published methods for testing TH perturbations in mice. In our experiments, however, we had to significantly alter our initial approaches. Although the exact causes of these discrepancies are unclear, closer examination of published reports reveals a wide range of reported treatment conditions, including variations in dose, length of treatment, mode of treatment delivery, the forms of TH used, and the ages, sex, and genetic strains of the mice. Similarly, outcome measures, including changes in circulating TH, vary widely due to these differences, underscoring the need to confirm parameters rigorously.

Given the inconsistencies from prior studies and the findings in our preliminary work, we set out to systematically test our treatment conditions. Although many reports focus solely on one sex [20–22], we assessed both males and females. We determined that levothyroxine in drinking water (20 *µ*g/mL) and a combination of two antithyroid medications (PTU in chow and methimazole in drinking water) over at least a two-month period was sufficient to induce changes in serum TH (Figure 2(a)). We evaluated serum and brain changes associated with TH manipulations. Mice provide critical experimental models and thus have been used extensively to study the effect of thyroid dysfunction on everything from cardiac function to cancer and to tease apart intracellular signalling mechanisms and gene regulation [20–23].

### 4.2. Physiological Changes due to TH Perturbation

TH levels are maintained in a relatively narrow range in peripheral tissues. When circulating levels of T4 change, most tissues adapt to maintain intracellular hormone concentrations in the homeostatic range [2, 3, 28, 29]. Increases in T4 levels, due to Graves disease or a thyroid-stimulating tumor, forces tissues to reduce influx and usage of the hormone, while stimulating enzymatic inactivation. Conversely, hypothyroidism induces changes necessary to conserve and utilize the available TH. Management of available T4 and T3 levels involves fine manipulation of transporter and enzyme levels at both transcriptional and posttranslation levels [6, 28, 30]. Our data from the adult mouse brain are in agreement with these prior reports.

We observed that the mouse brain adapts strongly to serum TH level perturbations to preserve the brain levels of T3 and T4 (Figure 2(b)), although the amount of T4 present in the brain was proportional to that circulating in the blood (Supplementary Figures S2A and S2B). Despite the homeostatic compensations, there were clear physiological effects in mice with altered T4 levels. This manifested through changes in blood glucose (Figure 3(e)), general activity levels (Figures 4(c)and 5(c)), and heart weight (Figure 3(f)). The decrease in body weight seen in humans with hyperthyroidism only occurred in the mice after three months of treatment (Figures 3(a)and 3(b)), which coincided with a substantial increase in both food and water consumption (Figures 3(c)and 3(d)). On the other hand, hypothyroidism in humans generally leads to increased lethargy, decreased activity, and weight gain. PTU/Met-treated mice dropped weight immediately upon treatment start (Figures 3(a)and 3(b)), then leveled off after a week and was amintained at that level throughout the study. While there are reports of PTU treatment causing weight loss in rodents [31], others report weight gain under similar conditions [32], possibly due to differences in animal age, drug dose, and length of treatment [33, 34]. TH regulation of metabolism is complex and multisystemic, involving the hypothalamus-pituitary axis as well as other tissues, such as the liver, pancreas, and adipose [6]. Although we did not tease apart the origins of the metabolic dysregulation in our model, it is likely that the TH-treated mice were hypermetabolic, with measurable hyperphagia concomitant with weight loss and a decrease in blood glucose.

PTU/Met-treated mice display decreased activity by two months of treatment (Figures 4(c)and 5(c)), consistent with human hypothyroidism. The other major cognitive domain that we tested was anxiety. During both the open field and elevated plus maze tests, the thyroxine-treated mice displayed decreased anxiety and increased exploration, spending more time in the center of the open field arena and on the open arms of the elevated plus maze (Figures 4 and 5). There was no concomitant increase in overall activity during either test. PTU/Met-treated mice exhibited low activity overall, which could account for the reduction in occupation of the open arms. Our data are consistent with published reports, although most draw the conclusion that hyperthyroid rodents display increased exploratory behavior due to increased anxiety, which is reflective of hyperthyroid humans [35, 36]. Although these behavioral change are intriguing, it is important to be careful extrapolating the results to human behavior. Indeed, time spent in the center of the open field arena or the open arms of the elevated plus maze could be a factor of physical or locomotor changes unrelated to anxiety.

There was significant mortality among the thyroxine-treated mice but only at the end of the treatment period (Supplementary Figure S3). Elevated circulating T4 led to large changes in systemic metabolic parameters, as evidenced by significant weight loss despite increased food consumption. Several animal fatalities occurred during the week of endpoint behavioral testing and, therefore, may have been induced by stress. Hyperthyroidism in humans, if left unchecked, is associated with increased mortality due primarily to cardiovascular symptoms, such as heart attack, stroke, hypertension, and increased heart rate leading to atrial fibrillation [37, 38]. In line with those clinical observations, we found substantially enlarged hearts in T4-treated mice (Figure 3(f)) and speculate that the deaths in that group were due to cardiac factors.

### 4.3. Regulation of Brain TH Levels

**Figure 7:**
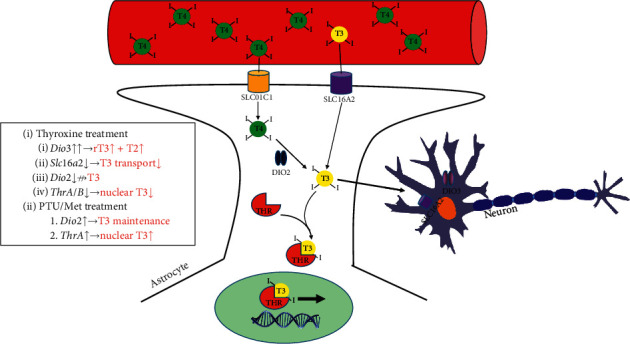
A model for the brain changes in response to hyper- and hypothyroidism. T4 circulates at a much higher concentration than T3. T4 crosses the blood-brain barrier via the astrocytic SLC01C1 transporter (OATPC1), whereas T3 enters via SLC16A2 (MCT8). T3, either transported from blood or converted from T4 by DIO2, complexes with TH receptors (THRA and THRB) in order to enter the nucleus and affect downstream gene expression. Additionally, T3 is delivered to neurons from astrocytes in order to regulate gene expression in those cells. DIO3 serves to inactivate TH (by converting to rT3 and T2). *Inset box*: Summary of measured changes (in black) and their theorized downstream effects (in red) that may help to maintain homeostatic TH levels in the brain during treatment. Our data indicate that, in response to thyroxine treatment, *Dio*3 mRNA expression is robustly increased, which would increase inactivation of TH. On the other hand, *Dio*2 expression decreases, which could prevent the activation of T4 to T3. *Slc*16*a*2 expression trends toward a decrease, indicating a possible reduction in transport. *Slco*1*c*1 expression is unchanged. The expression of the TH receptors decreases, reducing the potential for aberrant regulation of gene expression. The changes observed due to PTU/Met treatment are less robust, although both *Dio*2 and *ThrA* expression trends toward an increase.

There are a few key nodes that can be regulated in order to minimize changes in tissue TH concentration (Figure 7). The glial transporter, SLC01C1 (OATPC1), is a primary known route by which T4 enters the brain [29, 39], although its gene expression was unchanged in both treatment groups (Figure 6(d)). Similarly, the expression of the other major TH transporter, *Slc*16*a*2 (MCT8), only showed a weak trend toward a decrease in thyroxine-treated mice (Figure 6(c)). Overall, suppression of TH transporter genes' mRNA expression did not appear to be a significant mechanism for maintaining TH levels in the brain.

Interestingly, the largest observed effect at the mRNA level was in the deiodinase enzymes that catalyze the transformation of T4 to the highly bioactive T3 (DIO2) or which convert T4 and T3 to their inactive products (DIO3). Brain *Dio*3 mRNA expression was particularly strongly stimulated in thyroxine-treated mice (Figure 6(b)). Conversely, *Dio*2 expression was moderately stimulated in PTU/Met-treated mice, particularly in the males (Figure 6(a); Table 1). Previous reports indicate that both deiodinases are robustly regulated at a posttranslational level [3, 14, 28]. In the case of *Dio*2, this primarily happens through ubiquitination and subsequent proteasomal degradation of the DIO2 protein, which has a short half-life (∼20 minutes) [3]. It is certainly possible that posttranslational control of activity and protein levels would be seen early in the treatment course. Collectively, these changes may help minimize fluctuations in intracellular T4 and T3 levels (Figure 7). Salient questions include whether the adaptive changes lead to other parallel cellular effects and whether perturbations in blood TH lead to other brain changes at the mRNA, protein, or functional level. Previous literature has shown that *Dio*3 expression, in particular, plays important developmental roles in brain morphology and function [40]. Interestingly, *Dio*3^*-/-*^ mice, which have elevated TH, exhibit multiple behavioral abnormalities, including reduced anxiety and depression [41]. Additional tests of neurocognition may shed light on the potential clinical consequences of these alterations.

### 4.4. TH Perturbations in the Aging Brain

Although the clinical implications of TH dysregulation in developing brain (“cretinism”) has been extensively studied, there remains imperfect knowledge about the impact of TH disease in the aging brain. Although estimates vary, hypothyroidism and hyperthyroidism affect approximately 20% and 5% of elderly individuals, respectively [42]. Undiagnosed and subclinical TH dysregulation may also occur in a significant proportion of the elderly population, who are at a higher risk for associated comorbidities [43, 44]. Thus, TH dysregulation is a potentially large and underappreciated public health problem in this expanding demographic.

Prior literature has set out clear guidelines for the treatment of infant and juvenile TH perturbations, including genetic analyses and detailed thresholds for treatment [10, 11]. Guidance for the treatment of elderly patients is complicated by several factors, including cardiac problems and a predisposition to treatment-related (and aging-related) adverse events [10, 11]. Furthermore, TH dysregulation that occurs at a subclinical level often remains undetected and, hence, untreated but still may contribute to atrial fibrillation, coronary heart disease, and death [45, 46].

One of the strengths of this work is the use of middle-aged mice for our studies. Much of the literature focuses on the developmental exposure to TH perturbations [47, 48]. On the other hand, our experiments focused on TH dysfunction beginning at 14 months old without prior exposure to hyperthyroid- or hypothyroid-inducing agents, more closely mimicking adult-onset TH dysregulation. From our initial dosage experiments, it appeared that both young (two months) and middle-aged (12+ months) mice display similar changes in circulating T4. It is possible that there would be age-related difference downstream, however. Indeed, previous work has shown that changes in body weight, rotarod performance, and other behavioral measures is blunted in aged mice versus middle-aged one [33].

### 4.5. Sexual Dimorphism

Sex-specific differences in susceptibility to thyroid dysfunction has been well documented in humans [49, 50]. Although we used both sexes in our study, it is difficult to rigorously parse sex-specific differences. This is attributable to a reduction in power when we isolate data from a single sex (*n* = 5–8 vs. *n* = 13 per treatment group). Overall, the measured outcomes trended in the same direction for both males and females (Table 1), although there were some notable differences. For instance, both serum and brain T3 increased in PTU/Met-treated females but were unchanged or decreased in males. In addition, PTU/Met-treated females displayed decreased food and water intake, whereas the males did not. On the other hand, PTU/Met-treated males had larger transcriptional changes, at least in the genes examined, than the females in the same treatment group. Interestingly, the expression of *Slc*16*a*2 was more robustly affected in male mice of both treatment group*s* (Table 1). Recent literature examined the sexual dimorphism of systemic effects of TH perturbations in adult mice [33, 34]. These authors found that, over a nine-week treatment, hypothyroid female mice showed larger changes in food and water intake, consistent with our results [34]. There were also observed sex differences in the measured exploratory behavior and gene expression, although the latter varied substantially by the tissue examined (adipose, heart, liver) [34]. Our work may build upon this by focusing on gene expression changes in the brain.

## 5. Conclusions

In summary, we generated and tested a novel workflow for altered circulating TH levels in aged wild-type mice using treatments commonly used in humans. After blood TH level changes, the mouse brains had compensatory mechanisms to prevent tissue-level perturbations, including regulation of transporters, receptors, and activating/deactivating enzymes. Even with these mechanisms to achieve homeostatic normalization, the treated mice displayed activity level changes in neurobehavioral tests that may reflect altered anxiety, as well as systemic differences in metabolism and tissue weight.

## Figures and Tables

**Table 1:** Sex-specific changes due to treatment.

ASSAY/TEST	CONTROL		THYROXINE		PTU/MET	
	MALES	FEMALES	MALES	FEMALES	MALES	FEMALES
SERUM T4 (*µ*g/dL)	8.881 ± 1.144	8.79 ± 1.69	98.04 ± 12.59^A^	89.63 ± 6.62^A^	1.33 ± 0.5	1.41 ± 0.38
SERUM T3 (*µ*g/mL)	2.26 ± 0.47	3.05 ± 0.34	3.88 ± 0.85	3.29 ± 1.66	1.25 ± 0.18	4.55 ± 1.17
BRAIN T4 (fg/mg tissue)	768.3 ± 20.38	648.9 ± 25.8	885.1 ± 39	790.5 ± 62.9^A^	782.8 ± 33.1	676.8 ± 12.6
BRAIN T3 (fg/mg tissue)	18.51 ± 3.41	23.82 ± 4.03	33.45 ± 3.95	22.17 ± 5.06	16.3 ± 6.26	42.84 ± 12.41
HEART (mg)	216.4 ± 16	162 ± 6.4	320.8 ± 17.4^A^	285.4 ± 18.5^A^	165.8 ± 21.2	133.3 ± 9.8
FOOD (g)	3.1 ± 0.1	3.1 ± 0.06	7.2 ± 1.2	5.5 ± 0.2^A^	3.4 ± 1	2.3 ± 0.1
WATER (mL)	6.4 ± 1.3	6.3 ± 0.6	15.6 ± 4.4	11.9 ± 0.2^A^	6.9 ± 3.2	4.2 ± 0.7

*OPEN FIELD*						
Distance (cm)	3943 ± 270	4194 ± 279	3748 ± 268	4771 ± 165	1780 ± 312^A^	2729 ± 243^A^
Time in center (s)	35.1 ± 8.1	44.5 ± 16.6	70.5 ± 15.5	63 ± 17.4	37 ± 13.6^A^	36.8 ± 8.1

*EPM*						
Immobility (s)	159.8 ± 9.8	147 ± 11.9	158.1 ± 7.1	133.5 ± 5.2	227.8 ± 14.2^A^	179 ± 15.3
Open arms (s)	29.3 ± 5.8	42.9 ± 11.4	95.5 ± 18.2^A^	95.1 ± 6.9^A^	6.6 ± 2.3	20.2 ± 3.5

*qPCR (fold change)*						
*Dio*2	0.95 ± 0.13	1.06 ± 0.1	0.67 ± 0.2	0.55 ± 0.09^A^	1.56 ± 0.49	1.17 ± 0.12
*Dio*3	1.1 ± 0.06	0.89 ± 0.06	10.1 ± 1.92^A^	13.9 ± 3.63^A^	1.03 ± 0.05	1.11 ± 0.14
*Slc*16*a*2	1.08 ± 0.09	0.9 ± 0.16	0.74 ± 0.06^A^	0.92 ± 0.1	1.32 ± 0.1	0.89 ± 0.12
*Slc*01*c*1	0.88 ± 0.07	1.14 ± 0.1	0.95 ± 0.09	0.99 ± 0.05	1.06 ± 0.07	1.05 ± 0.09
*Thra*	1.06 ± 0.04	0.93 ± 0.07	0.78 ± 0.05^A^	0.72 ± 0.05^A^	1.23 ± 0.08	0.95 ± 0.03
*Thrb*	0.94 ± 0.07	1.08 ± 0.05	0.82 ± 0.05	0.92 ± 0.06	1.02 ± 0.09	0.96 ± 0.04
*Ttr*	1.02 ± 0.07	0.98 ± 0.05	0.81 ± 0.06	0.89 ± 0.06	1.18 ± 0.06	0.92 ± 0.06

^a^ *p* ≤ 0.05compared with control.

## Data Availability

In order to access supporting data, contact the corresponding author.

## Supplementary Materials

Supplementary Materials The Supplementary Material contains two tables and three figures. Supplementary Table S1 is an overview of the mice used for these experiments, while Supplementary Table S2 gives details of the gene expression assays used. Supplementary Figure S1 shows the levothyroxine dosage experiments in young mice (A-B), as well as a short (two week) treatment in middle-aged mice (C). Supplementary Figure S2 shows the correlation between serum and brain T4. Supplementary Figure S3 is the survival curve for the four month treatment in middle-aged mice.
