# Supplementary material for: Modulating Thyroid Hormone Levels in Adult Mice: Impact on Behavior and Compensatory Brain Changes
Source: J Thyroid Res. 2021 Jun 24;2021:9960188. doi: 10.1155/2021/9960188 (PMC8253651; doi:10.1155/2021/9960188)
Supplement: Supplementary Materials — The Supplementary Material contains two tables and three figures. Supplementary Table S1 is an overview of the mice used for these experiments, while Supplementary Table S2 gives details of the gene expression assays used. Supplementary Figure S1 shows the levothyroxine dosage experiments in young mice (A-B), as well as a short (two week) treatment in middle-aged mice (C). Supplementary Figure S2 shows the correlation between serum and brain T4. Supplementary Figure S3 is the survival curve for the four month treatment in middle-aged mice. [file 9960188.f1.docx]

**Supplementary Table S1: Overview of Animal Numbers and Ages**

| Assay/Test | # Animals (C/T/P) | Starting Age | Treatment  Length at test | Figure |
| --- | --- | --- | --- | --- |
| serum th | 6/6 | 2-6 months^a^ | 2 weeks | S1B |
|  | 6/7/7 | 13 months | 2 weeks | S1C |
|  | 13/13/13 | 14 months | 2 months | 2A, S3A |
|  | 13/13/13 | 14 months | 4 months | 2A, S3A |
| brain th | 13/13/13 | 14 months | 4 months | 2B, S3B |
| weight | 13/13/13^b^ | 14 months | 4 months | 3A,B |
| food/water | 13/13/13 | 14 months | 3 months | 3C,D |
| blood glucose | 13/13/13 | 14 months | 3 months | 3E |
| heart weight | 13/10/13 | 14 months | 4 months | 3F |
| open field | 13/13/13 | 14 months | 2, 4 months | 4B,C,D |
| epm | 13/13/13 | 14 months | 4 months | 5B,C,D,E |
| gene expression | 13/13/13 | 14 months | 4 months | 6A-G |
|  |  |  |  |  |

^a^ See Supplementary Figure 1A for an explanation of the age range

^b^ Two mice died in the last week of the study and were not included in the final week’s average for the thyroxine group

**Supplementary Table S2: Gene-Specific Taqman Assays**

| **Gene** | **Gene Name** | **Ref. Seq.** | **Assay ID** |
| --- | --- | --- | --- |
| **Pgk1** | Phosphoglycerate kinase 1 | NM_008828.3 | Mm00435617_m1 |
| **Sdha** | Succinate dehydrogenase subunit A | NM_023281.1 | Mm01352366_m1 |
| **Dio2** | Deiodinase type II | NM_010050.2 | Mm00515664_m1 |
| **Dio3** | Deiodinase type III | NM_172119.2 | Mm00839358_s1 |
| **Slc16a2** | Solute carrier family 16, member 2 | NM_009197.2 | Mm00486204_m1 |
| **Slc01c1** | Solute carrier organic anion transporter family member 1c1 | NM_021471.2 | Mm00451845_m1 |
| **Thra** | Thyroid hormone receptor alpha | NM_1780060.3 | Mm00579691_m1 |
| **Thrb** | Thyroid hormone receptor beta | NM_001113417.1 | Mm00437044_m1 |
| **Ttr** | Transthyretin | NM_013697.5 | Mm00443267_m1 |

**Supplementary Figure Legends**

Supplementary Figure S1. (**A**) Timeline of short-term dose-response levothyroxine treatments. Serum T4 was measured on the same cohort of mice, treated sequentially with 2, 3, 4, then 20 µg/mL levothyroxine, starting at 2 months old. (**B**) Though both 3 (p<0.02) and 4 (p<0.03) µg/mL thyroxine led to a significant increase in circulating T4 compared with control animals at each time point, only 20 µg/mL elicited a robust response (p<0.00004). (p<0.09 for 2 µg/mL) N= 3M/3F in each group. (**C**) 13 month old mice were treated with 20 µg/mL thyroxine, a combination of PTU/Met, or control chow and water. Although this dose of thyroxine led to a large change in circulating T4 by two weeks (p<0.01), treatment with PTU and methimazole did not lead to a significant change at this timepoint (p<0.27). In subsequent experiments, we lengthened the treatment period. Control = 3M/3F, Thyroxine = 4M/3F, PTU = 3M/4F.

Supplementary Figure S2. Middle-aged (13 months old) mice were treated with either levothyroxine (20 µg/mL) or a combination of PTU and methimazole for 4 months. Serum T4 was measured at both two and four months, while brain levels were measured at four months. Even though there was a difference in the sample millieu (serum vs. brain RIPA extract), and despite an apparent disconnect in the magnitude of change in the serum versus the brain in the treatment groups, there was a strong positive correlation between circulating and brain T4 at both two (**A**) and four (**B**) months. N=11-13 per group at each timepoint, split approximately equally between sexes.

Supplementary Figure S3. Survival curve for Control, Thryoxine, and PTU/Met-treated mice during the treatment course. Note that the survival curves for Control and PTU/Met sit directly on top of each other as there were no deaths. Only the thyroxine-treated group had attrition, and that only occurred during the final week of treatment.


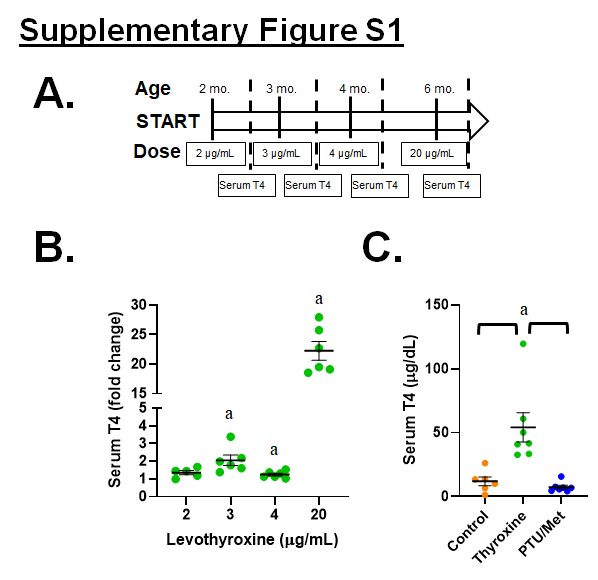


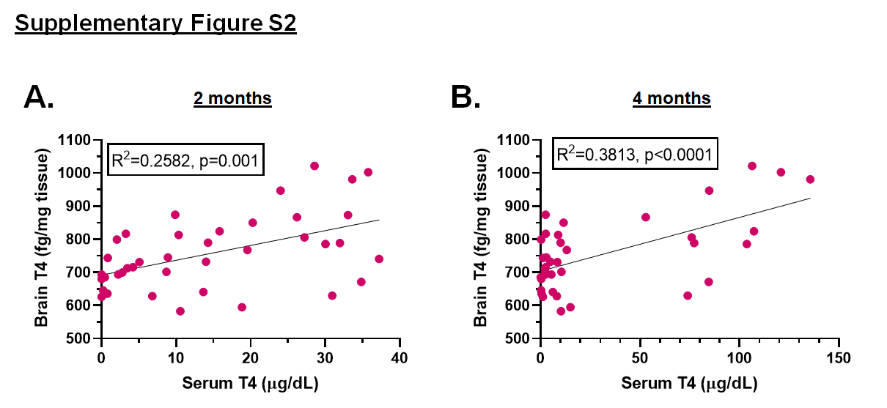

**Supplementary Figure S3**
